# Random Lasing for Bimodal Imaging and Detection of Tumor

**DOI:** 10.3390/bios13121003

**Published:** 2023-11-29

**Authors:** R. Gayathri, C. S. Suchand Sandeep, C. Vijayan, V. M. Murukeshan

**Affiliations:** 1 Centre for Optical and Laser Engineering (COLE), School of Mechanical and Aerospace Engineering, Nanyang Technological University (NTU), Singapore 639798, Singapore; gayathri.radhakrishn@ntu.edu.sg (R.G.); ssuchand@ntu.edu.sg (C.S.S.S.); 2 Department of Physics, Indian Institute of Technology Madras (IITM), Chennai 600036, India; cvjiitm@gmail.com

**Keywords:** random lasing, tumor, early detection, imaging, spectroscopy, cancer

## Abstract

The interaction of light with biological tissues is an intriguing area of research that has led to the development of numerous techniques and technologies. The randomness inherent in biological tissues can trap light through multiple scattering events and provide optical feedback to generate random lasing emission. The emerging random lasing signals carry sensitive information about the scattering dynamics of the medium, which can help in identifying abnormalities in tissues, while simultaneously functioning as an illumination source for imaging. The early detection and imaging of tumor regions are crucial for the successful treatment of cancer, which is one of the major causes of mortality worldwide. In this paper, a bimodal spectroscopic and imaging system, capable of identifying and imaging tumor polyps as small as 1 mm^2^, is proposed and illustrated using a phantom sample for the early diagnosis of tumor growth. The far-field imaging capabilities of the developed system can enable non-contact in vivo inspections. The integration of random lasing principles with sensing and imaging modalities has the potential to provide an efficient, minimally invasive, and cost-effective means of early detection and treatment of various diseases, including cancer.

## 1. Introduction

Biological tissues are considered as disordered photonic structures because they contain complex and irregular arrangements of cells and tissues, which lead to diffusive light scattering [1,2]. This diffusive scattering results in an increased dwell time of light within the tissues and can be utilized as the feedback mechanism required for obtaining lasing emission in the presence of an appropriate gain medium [3]. This unconventional method of achieving lasing through multiple scattering induced by the inhomogeneities in the medium is termed as random lasing. The strength of light localization and lasing feedback is primarily determined by the degree of disorder in the medium. The emission characteristics, such as lasing threshold, linewidth, peak position, and intensity, are highly sensitive to changes in the disorder [4,5]. Random lasing thus has the potential to offer insights into the scattering dynamics and light propagation in disordered media such as biological tissues [6].

In 2004, Polson et al. pioneered the demonstration of random lasing emission in biological tissues and, since then, it has been realized in various biological tissues including human organs, such as the brain, breast, thyroid, etc. [3,7,8,9,10]. Being highly sensitive to disorder, random lasing has been demonstrated to be useful in detecting even nanoscale structural changes and deformations in bones [11]. A recent study has also shown that random lasing emission can be used to differentiate different types of tissues, such as fat, nerves, muscle, and skin, even under normal room light conditions [12]. Recently, Wang et al. successfully demonstrated random lasing in tissues that had been impregnated with an organic dye [13]. These findings provide the scope for using random lasers in various biomedical applications such as in vivo diagnosis and monitoring of diseases, tissue differentiation during laser surgeries, human antibody sensing, and so on. The use of fluorescent anticancer drug molecules that bind to intracellular targets like estrogen receptors to achieve random lasing holds great promise for photodynamic therapies [14].

Cancer is unarguably one of the leading causes of death worldwide and much interest was generated when random lasing signals showed potential in distinguishing cancerous tissues from healthier ones [13]. Random lasing emission from cancerous tissues was found to contain more laser lines than healthier ones, arising from the increased number of laser resonators brought on by the increased disorder in cancerous tissues. The power Fourier transform of the emission spectra provides information about the cavity length of the laser resonator in the tissue, facilitating the mapping of cancerous regions [15]. By analyzing the lasing parameters, like lasing threshold and modes of lasing, it is also possible to determine the malignancy grade of the cancer, offering a valuable tool for tissue classification in diagnostic applications [13]. When cancer is diagnosed at its early stages, the chances of survival are very high [16,17]. In the early stages of cancer, the abnormal growth is referred to as a primary tumor. Tumors can either be benign or cancerous [18]. A cancerous tumor will grow further and spread to other parts of the body, leading to fatal health conditions. The size of the tumor is one of the important prognostic factors to determine its malignancy potential and is inversely related to the chance of survival [18]. Larger tumors are more likely to develop into cancer. Though smaller tumor polyps are likely to be benign, some of them grow over time to become adenomatous or cancerous, and it is difficult to determine whether it becomes so without performing a biopsy [19]. In the case of solid tumors, for example colorectal tumors, which develop in the inner linings of the colon or rectum, polyps with a size greater than 2 cm have approximately a 40% risk of being cancerous [20]. Hence, it is advised to remove even smaller polyps to eliminate the risk from such growth. The detection of such smaller polyps will enable the early diagnosis of tumors. However, polyps of millimeter sizes are difficult to identify, even using state-of-the-art techniques like colonoscopy. Hence, several techniques are being developed to study the morphological, biochemical, and biomechanical changes in tumor regions and their microenvironments [21,22,23,24,25]. It has been found that tumor progression causes changes in the local refractive index, biomechanical properties, surface roughness, and viscoelasticity of the tissues [26,27,28]. Changes in these properties will modify the scattering scenario and thus can be detected using random lasing. 

Together with early detection, high-resolution imaging of tumor regions is equally important for cancer diagnosis and treatment. A technique incorporating sensitive detection and concurrent imaging will facilitate image-guided feedback for diagnosing and monitoring cancer growth. The high photon degeneracy and low spatial coherence of random lasers make them appealing for imaging applications. Recent research reports show that random lasers could generate speckle-free images even in highly scattering environments [29,30,31]. Random lasers have been used for high-contrast in vitro dental imaging in the backscattering configuration to image through turbid media, for imaging dynamic phenomena like the blood flow patterns in mouse ear skin, and for other imaging applications [32].

Recent research demonstrated how surface corrugations on polymer substrates affect random lasing [33]. Distinct features were observed in the lasing spectra, threshold, and linewidth characteristics with surface roughness variations. Random lasing studies based on such morphological and surface-level variations in tissues pave the way to the development of novel and minimally invasive diagnostic tools. In this context, we propose a random laser-based diagnostic technique comprising the imaging and sensing capabilities of random laser to create a bimodal interrogation system for the precise and early-stage detection of tumors. Here, we focus the investigation on random lasing characteristics that are dependent on surface-level variations associated with tumor progression. The initial investigation of the proposed method is conducted by theoretically modeling tumorous tissues and exploring the influence of surface corrugations on its light transport properties, followed by experimental validation on a phantom sample. A bimodal spectroscopic and imaging system is designed based on the random lasing principles. The lasing emission from the dye-impregnated phantom tissue is concurrently utilized as an illumination source to image the tumor and for spectral analysis. The instantaneous spectral feedback and image guidance will assist in the precise identification of the surgical margins, and hence, this technique will have huge potential, not only for cancer diagnosis, but also for the realization of minimally invasive, image-guided, therapeutic laser surgeries for cancer removal in the future.

## 2. Materials and Methods

### 2.1. Theoretical Modelling

The scattering and light localization properties in healthy and tumorous tissues were theoretically investigated using the finite-difference time-domain (FDTD) numerical simulation method. A commercial software package, Ansys Lumerical, which is a 3-dimensional (3D) Maxwell’s electromagnetic equation solver based on FDTD, was employed for the simulation of the structures and theoretical analysis [34]. A biological tissue model of the inner layer of the colon was simulated using the refractive index data of mucosa [35]. This is particularly relevant because tumors typically originate from the epithelial layer of the mucosa. Furthermore, the mucosa is the tissue that is directly observed during endoscopy [36]. Due to computational limitations, a smaller section of the tissue, measuring 1.5 µm × 1.5 µm × 50 µm, was simulated. However, a perfectly matched layer (PML) boundary condition was employed to define the simulation domain on all sides. This minimized light reflections at the boundary by efficiently absorbing the incident light. A non-uniform conformal variant 0 meshing was employed in the simulation for improved accuracy and better resolution of interfaces. 

**Figure 1:**
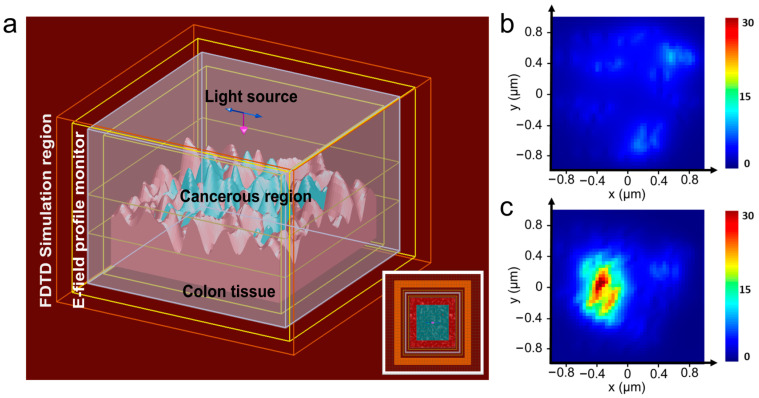
(**a**) The simulation window showing the 1 µm × 1 µm sized tumorous region in healthy colon tissue. The tissue is illuminated using a TFSF source above and the E-field profile monitor analyzes the electric field distribution. The inset shows the top view. (**b**) The normalized electric field intensity profile at the surface of the healthy tissue model and (**c**) the normalized electric field intensity profile at the surface of the model with simulated tumorous tissue.

Based on refractive index data obtained from healthy colon tissues, the real part of the refractive index at the wavelength of interest (570 nm) was 1.3439 [35]. With the progression of tumors, there is an observed increase in the refractive index. The refractive index contrast ($\Delta n=n_{t}-n_{h}$, where $n_{t}$ represents the refractive index of the tumor region and $n_{h}$ represents that of the healthy region) at the 570 nm wavelength was approximately 0.015 for both the high-grade tubulovillous adenoma (a pre-cancerous polyp with heightened risk of malignant transformation) and for the low-grade adenocarcinoma (cancerous polyp) that develops in the tubulovillous background [35]. Therefore, in order to simulate the tumorous region, a flat polyp of 1 µm × 1 µm size was realized within the healthy tissue region with the refractive index value set to 1.3589. The mesh order for the tumor region was configured as 1, while for the healthy region, it was set to 2. This is important because materials with a lower mesh order are given precedence over those with a higher order when overlapping regions are meshed in the simulation. In comparison to healthy tissue regions, tumorous regions are reported to exhibit a two-fold increase in the height of both micro- and nano-scaled structures [37]. Additionally, there is a two-fold elevation in the total surface area to volume ratio and porosity. Consequently, for simulation purposes, the root mean square (rms) amplitude of surface irregularities was set to 15 µm for the healthy region and 30 µm for the tumorous region. The 3D simulation setup is shown in Figure 1a. The inset shows the top view of the tissue surface with the simulated cancerous tissue region in the center (shown in blue shade). 

The background refractive index of the simulation region was set to be 1. A total-field/scattered-field (TFSF) source with emission centered at 570 nm with a 100 nm span was used to illuminate the tissue surface. While the actual experiment utilized a Gaussian laser beam, it is worth noting that due to the significantly smaller size of the simulated region in comparison to the interrogation area, it is safe to approximate the illumination to be a plane wave. Moreover, it is common practice to use TFSF sources in simulations for random and lossy structures. A 3D frequency-domain field and power monitor was placed in such a way that it covered the entire surface. The field profile monitor provides information on the light propagation and localization at the surface. The simulation was carried out both with and without the presence of the tumorous region for comparative analysis. Figure 1b and 1c show the simulation results of the electric field distribution on the healthy tissue model and on the model with the tumor growth, respectively. The maximum electric field intensity (normalized to the incident field intensity) recorded on the healthy tissue was 9.37, whereas it was 34.2 at the tumor region. Evidently, the electric field localization is 3.6 times stronger on the cancerous tissue as compared with the healthy tissue. The increased surface roughness and corrugations in the tumor region promote more optical scattering within the surface plane, leading to the strong localization of light. This enhanced localization can be harnessed to provide better feedback for random lasing. Consequently, due to the sensitivity of random lasing properties to light localization, these variations in surface structures can indeed impact the lasing parameters.

### 2.2. Phantom Tissue Model of Tumor Polyp

**Figure 2:**
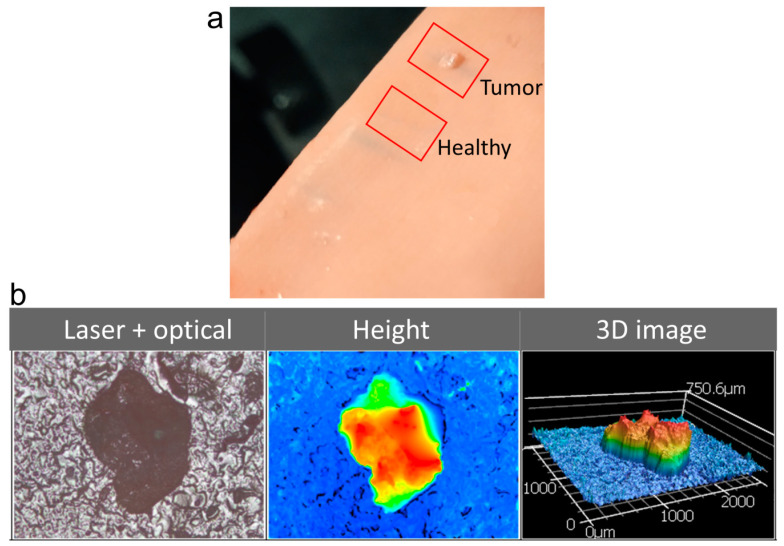
(**a**) Photograph of the phantom tissue with the healthy and tumor regions marked. (**b**) The optical microscopic image, surface height profile, and 3D image of the tumor growth simulated on the tissue surface recorded using a laser scanning confocal microscope. The color represents the height map.

A phantom tissue is a simulated tissue that resembles and mimics the properties of real tissues. For a proof-of-concept demonstration of the proposed idea, a commercial phantom tissue sample purchased from Simulab Corporation, Seattle, WA, USA was used for the investigations. The early-stage tumor was realized on the phantom tissue sample as a millimeter-sized polyp growth with higher surface roughness. The tumor polyp created had an area of approximately 1 mm^2^ and a thickness of 0.5 mm, which is similar to the initial tumor growth stages. Figure 2a shows the actual photograph of the phantom tissue with the regions under investigation highlighted. Figure 2b shows a microscopic image of the tumor polyp, the height profile, and a 3D image, which were recorded using a laser scanning confocal microscope (VK-X1000, Keyence (Osaka, Japan), 5× magnification, 0.13 NA). The surface roughness (S_a_) value was measured to be 14 µm for the healthy tissue region and 35 µm for the tumor region. 

**Figure 3:**
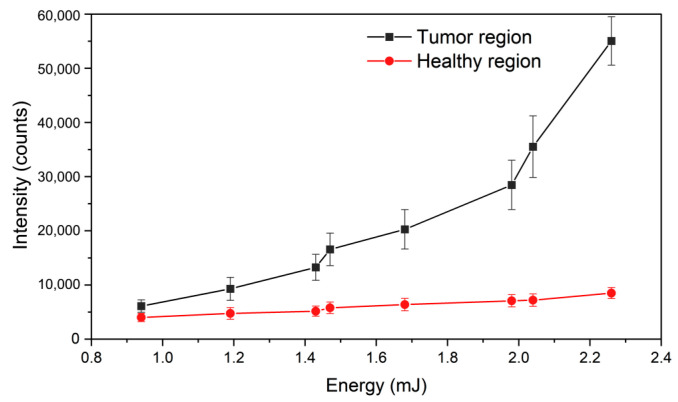
Emission intensities from tumorous and healthy regions with increasing pump energies.

The gain medium for random lasing was prepared by dissolving 6 mg of rhodamine 6G (R6G) in 1 mL dimethyl sulfoxide (DMSO) and the tissue samples were soaked in it. It should be noted that the R6G gain medium used for this proof-of-concept demonstration is not suitable for live cell imaging. In real tissue samples, biocompatible fluorescent anticancer drugs and organic dyes could be used to generate random lasing [13,14]. The phantom tissue sample was initially investigated for lasing using a standard random lasing experimental setup [33]. 532 nm second harmonic emission from an Nd:YAG laser (VIBRANT 355 II, OPOTEK Inc., Carlsbad, CA, USA) running at 10 Hz repetition rate and 5 ns pulse duration was used as the excitation source. The laser beam was focused using a cylindrical lens (f = 7 cm) onto the sample. The emission spectra and intensities from the healthy and tumor tissue regions with increasing energies of the pump laser were recorded using a snapshot fiber optic spectrometer (USB 4000, Ocean optics, Orlando, FL, USA) and are shown in Figure 3. The simulated tumor region showed a distinct threshold in the emission at around 1.95 mJ pump energy. A rapid increase in the intensity, as well as linewidth narrowing to 7 nm were observed beyond this threshold. On the other hand, such a lasing behavior was not observed from the healthy regions, even at much higher pump energies, up to 5.3 mJ. 

It is important to emphasize that the tumor polyp possesses a refractive index identical to the healthy region in this measurement. Consequently, the observed emission behavior is solely attributed to the influence of surface corrugation. Any changes associated with the refractive index contrast are not factored into this consideration [38,39,40]. In reality, however, the refractive index contrast will also be a contributing factor, potentially further enhancing the lasing behavior.

### 2.3. Random Laser Based Bimodal Spectroscopic and Imaging System

**Figure 4:**
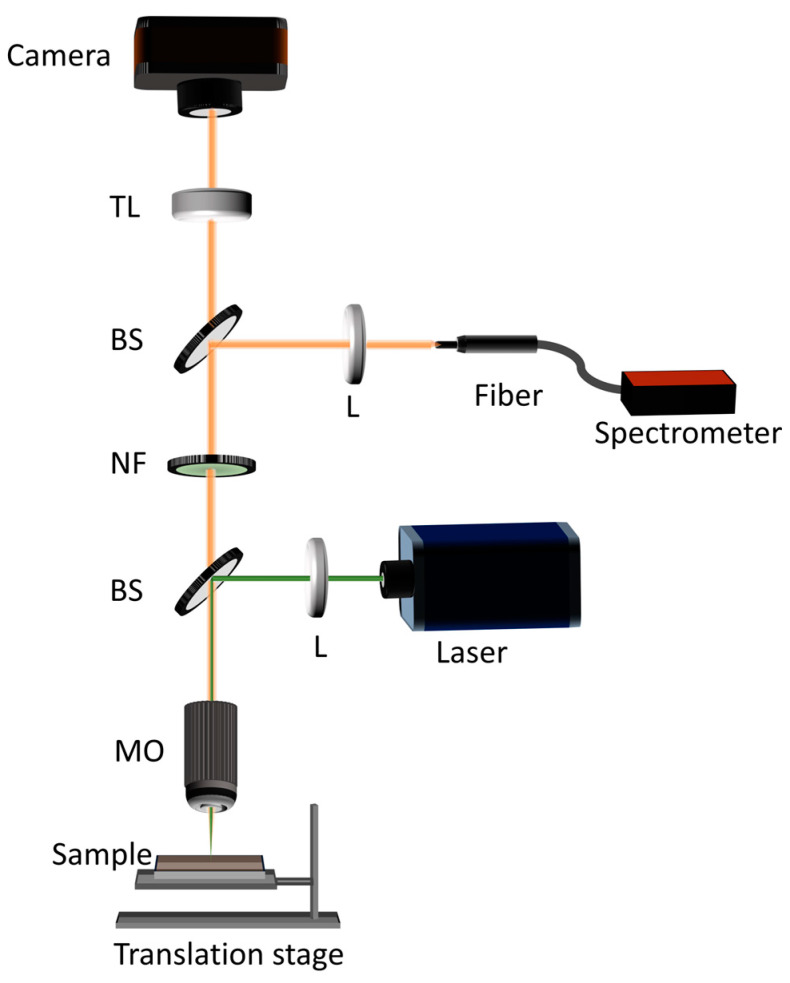
A schematic representation of the developed bimodal detection system. BS: beam splitter, MO: microscope objective, NF: notch filter, TL: tube lens, L: lens.

A bimodal system utilizing the random lasing emission from dye-impregnated tissues for spectral analysis and concurrent imaging of the tissue surface was designed and fabricated. The schematic representation of the experimental system developed is shown in Figure 4. The phantom tissue sample was mounted on an XYZ motorized translation stage (T-LS28M, Zaber Technologies, Vancouver, BC, Canada). The 532 nm pulsed laser emission from the Nd:YAG laser (VIBRANT 355 II, OPOTEK Inc.) was directed using a pellicle beam splitter (BP145B1, Thorlabs, Newton, NJ, USA) and focused using a 10× microscopic objective (Plan N, 0.25 NA, Olympus, Tokyo, Japan) onto the tissue sample. The large working distance (10.6 mm) of the objective, enabled non-contact inspection. A plano-convex lens with a focal length of 7.5 cm was placed in the beam path before the pellicle beam splitter to decouple the focusing plane and imaging plane. This ensured a larger illumination area at the imaging plane. The fluorescence emission from the sample was collected using the same microscope objective and was directed to a tube lens (TTL 200, Thorlabs) through the pellicle beam splitter. A notch filter centered around 532 nm (NF 533-17, OD 6 at a center wavelength of 533 ± 2 nm, Thorlabs) was employed in the imaging path to eliminate the 532 nm pump beam reflected and scattered from the sample. The images were recorded using a scientific complementary metal oxide semiconductor (sCMOS) camera (Neo 5.5, Andor, Belfast, UK). To monitor the spectral response, a part of the emission beam was directed to the fiber optic head of the handheld snapshot spectrometer (USB 4000, Ocean optics) using another pellicle beam splitter (BP145B1, Thorlabs), as shown in Figure 4. 

The resolution of a microscopic imaging system holds utmost significance for its intended applications. In general, the spatial resolution of a wide-field microscopic system is inherently constrained by diffraction. The theoretical resolution limit of a wide-field imaging system is given by 0.61*λ*_i_/*NA*_i_, where *λ*_i_ is the wavelength of the light and *NA*_i_ is the numerical aperture of the objective lens used for imaging [41]. In the current imaging configuration, which used a microscopic objective with an NA of 0.25, collecting fluorescent emission at a wavelength of around 560 nm, the theoretical resolution limit is calculated to be 1.4 µm. It is imperative to account for the resolution of the camera sensor as well, which is primarily determined by the pixel size of the sensor. The Nyquist resolution limit for an image sensor is defined as 2 times the effective pixel size, expressed as 2× pixel size/magnification. In the case of the sCMOS camera used in the system, the sensor consisted of an array of 2560 × 2160 pixels, with each pixel having a dimension of 6.5 µm × 6.5 µm. Paired with the 10× magnification objective, the Nyquist limit for this camera would be 1.3 µm, which is smaller than the theoretical optical resolution limit of 1.4 µm. This implies that the system developed is fully capable of achieving the theoretical spatial resolution limit. The spatial resolution of the microscopic imaging system was experimentally evaluated by imaging a standard 1951 USAF high-resolution test chart (2″ × 2″ negative test target, Edmund Optics, Barrington, NJ, USA). The system was capable of resolving group 8 element 3 of the test chart, corresponding to a spatial resolution of 1.55 μm, with an area of interrogation of 1.6 mm × 1.4 mm in a single frame.

## 3. Results and Discussion

### Image and Spectral Acquisition Using the Bimodal System

**Figure 5:**
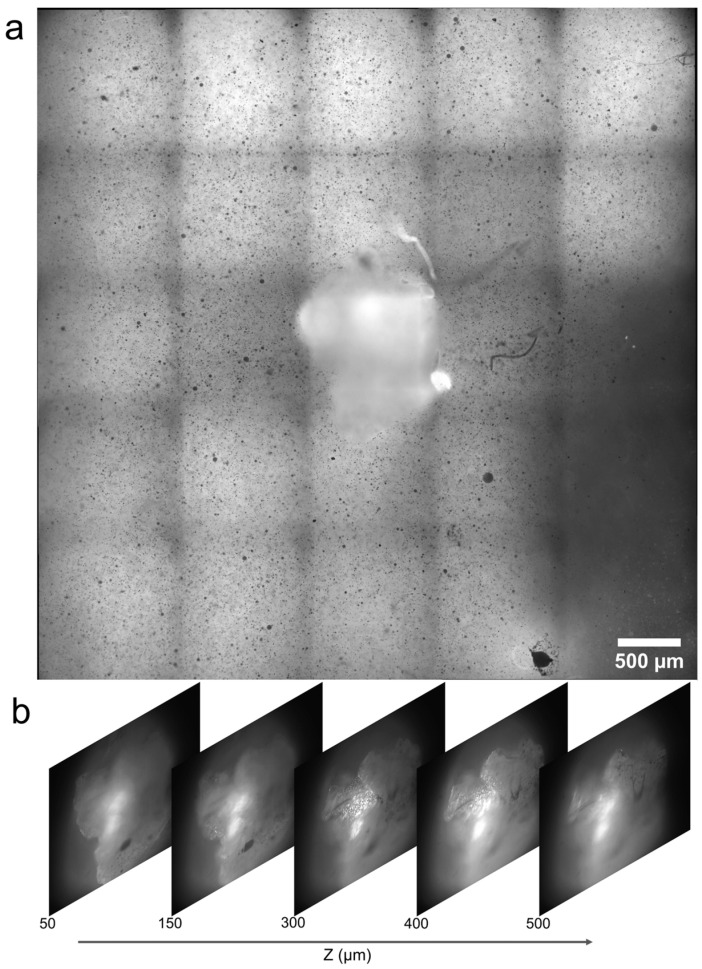
(**a**) Image of the phantom tissue surface containing the simulated tumor obtained by stitching the individual frames recorded. (**b**) Images recorded at different Z-planes of the tumor.

The phantom tissue sample containing the simulated tumor growth was excited with 532 nm laser pulses focused to an area of approximately 1.6 mm × 1.4 mm using the microscope objective. The image/signal acquisition time of the sCMOS camera and spectrometer were set to 0.1 s. Images were recorded in the fluorescence imaging configuration, which is one of the most commonly used imaging modalities in biomedical imaging [42,43,44,45]. The sample was translated in 1 mm steps in the X- and Y-directions using the motorized translation stage. Images, as well as the spectral data of the sample were recorded at each step location. The recorded images were stitched together using a commercial scientific image analysis software, Image-Pro^®^ Premier (v 9.0), and the final stitched image generated is shown in Figure 5a. As the imaging plane was set to the phantom tissue surface, the upper surface of the tumor appears defocused. Nevertheless, the boundaries of the tumor are clearly visible in the image recorded. Images were also captured by translating the sample in the Z-direction, recording different planes of the tumor. Figure 5b shows the images recorded from different Z-planes. The corresponding Z-distances are marked in the figure, considering the phantom tissue surface to be the Z = 0 plane.

**Figure 6:**
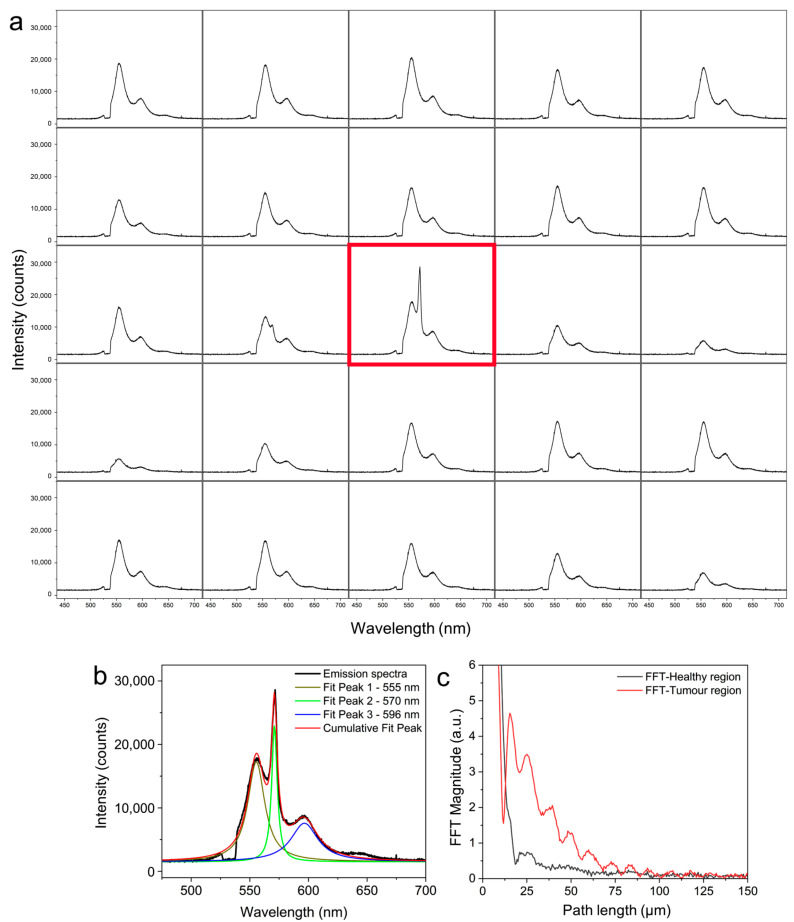
(**a**) Emission spectra recorded at each frame corresponding to the image shown in Figure 5a. The spectrum obtained at the tumor region is highlighted in red. (**b**) Deconvoluted spectral components of the emission spectra obtained from the tumor region. (**c**) Power Fourier transforms of the emission spectra from the healthy and tumor regions.

The image depicted in Figure 5a was generated by stitching 25 (5 × 5) images. The spectral data at each of these locations were also recorded simultaneously using the snapshot spectrometer. The recorded spectra are shown in Figure 6a in the same sequence of the images shown in Figure 5a. The emission spectrum corresponding to the tumor region is highlighted in red in Figure 6a. 

While healthy tissue regions emit normal fluorescence, the presence of higher disorder in the tumor region leads to the emergence of a distinct lasing peak on top of the fluorescence emission. Figure 6b shows the deconvoluted emission peaks corresponding to the emission spectrum in the tumor region, revealing the random lasing emission centered at 570 nm with a linewidth of 5 nm. The power Fourier transform (PFT) of the emission spectrum is analyzed to deduce the path length of the photons undergoing scattering [15,33]. Figure 6c shows the computed PFT spectra of the emission from both the healthy and tumor regions (shown in black and red colors, respectively). The first peak in the PFT corresponds to the path length of the stable resonant mode within the random cavity, which was evaluated to be 15.4 µm for the tumor region. In contrast, the PFT from the healthy region is devoid of stable resonant paths, which explains the absence of lasing emission from the healthy tissues. These results indicate that the corrugations on the tissue surface provide the necessary cavity feedback for lasing, and changes to the morphological and surface properties can affect the light localization and lasing emission. As random lasing measurements provide vital information about the cavity length and degree of disorder in the tissues, they may even be used for categorizing the cancer based on its malignancy grade [15]. In addition to the surface variations, the disorder developed within the bulk of the tissues during tumor growth can also contribute to the random lasing and further enhance the emission characteristics [13]. Therefore, the random lasing parameters from the tissues could be utilized to interrogate the changes in the tissue characteristics and tumor detection. 

The concept of this bimodal detection system using random lasing principle utilizes the emission from dye-impregnated tissues as a light source for imaging and for sensing the tumorous regions. The capability to image the tissues along with spectral feedback can be of utmost importance for early diagnosis. Another advantage of this technique is that it does not require selective staining of tumor regions, unlike other fluorescence-based diagnostic methods, as the detection in this scheme is based on the inherent roughness variation in the tumorous tissues. The capability of the random lasing illumination source for artifact-free imaging in scattering environments is an added advantage of the proposed method. Recently, there has been a surge in the adoption of artificial intelligence (AI) assisted techniques in the field of bio-diagnostics, which offer cost-effective, scalable, and efficient solutions [46,47]. In the context of colon cancer detection, AI-based methodologies are employed in biomarker screening, gene detection, histopathologic classification, etc. [48,49]. By leveraging the images and spectra captured by the random laser based bimodal system, intelligent analytical methods can be applied for precise identification and early-stage diagnosis. This integrated approach could offer a comprehensive and multi-modal strategy, holding the potential to significantly enhance diagnostic accuracy, revolutionizing traditional diagnostic techniques like colonoscopy.

## 4. Conclusions

A random laser-based technique to detect early-stage tumor growths based on the surface modifications of tumorous tissues is conceptualized, developed and illustrated. Initial investigations using numerical simulations explicitly indicate that the increased surface corrugations of tumor regions support strong localization of electric field energy. Experimental investigations carried out on phantom tissue samples showed that the spectral responses from the healthy and tumor regions are quite distinct. Significantly, the tumor region exhibited random lasing emission even at lower pump energies, while the healthy tissues continued to emit fluorescence signals. Analysis of the emission spectra through PFT calculations revealed the existence of stable resonant paths in the tumor region, attributed to the increased disorder. This higher degree of disorder is responsible for the observed lasing emission, which was notably absent in the healthy tissue region. Further, a bimodal diagnostic system incorporating imaging and spectroscopic detection modalities was designed and developed. The random laser emission from the dye-impregnated tissues provides the spectral signature for tumor detection and, at the same time, acts as an illumination source for imaging the tissues. Results from these investigations show that random lasing signals can be used to identify and image polyps as small as 1 mm^2^ and hence could facilitate early diagnosis of tumor growth. The provision of simultaneous imaging of the tissues along with sensitive detection can leverage the scope of random lasers for endoscopic applications. This also opens up novel possibilities for random lasing feedback-based visually guided and spectroscopically controlled automated laser surgery systems based on artificial intelligence for removing tumor growths.

## Data Availability

The datasets generated and/or analyzed during the current study are available from the corresponding author on reasonable request.
